# Are all Italian newborns equal?

**DOI:** 10.1186/1824-7288-40-S2-A9

**Published:** 2014-10-09

**Authors:** Stefano Semplici

**Affiliations:** 1 University of Rome “Tor Vergata”, Rome, 00173, Italy

## 

The debate on the restructuring of Chapter Five (Titolo Quinto) of the Italian Constitution has brought once again the issue of the allocation of powers between the State and its regions under the spotlight. According to Article 117 of the Italian Constitution, as reformed in 2001, the State and its regions share normative competences in regulating certain matters. In the field of core civil and social rights, including health care, it is the responsibility of the State to identify fundamental levels of services and assistance that have to be guaranteed to everyone across the nation. The aim of this provision was to prevent unacceptable disparities in the enjoyment of the right to health. In practice, however, this has not been the case. On the one hand, Italian regions have disciplined certain matters in fairly different ways, forcing the Ministry of Health to exercise its role of warrantor of fairness more than expected. On the other hand, the quality of services has been greatly affected by a “geographical” factor, depending on availability of resources, different organization of health system and bureaucracy at regional level.

Health care services for newborns are a case in point. Two examples – among others – are worth exploring further. The neonatal screening programmes for cystic fibrosis and inherited metabolic diseases are a lot different among different regions and sometimes even among different towns or hospitals in the same town. Some legislative measures have been taken to overcome this situation, but the implementation of the national regulation remains difficult to attain. Should the responsibilities and decisions involved depend on the place a baby is born? As a matter of fact, this observation keeps playing an important role as to the possibility of addressing the most critical situations in a successful way. Even though the neonatal mortality rate in Italy is nowadays among the lowest in the world, infants born in Southern Italy still run a higher risk of dying in the neonatal period. Against this background, the situation of neonatal intensive care units points to inequalities that are not new for the country and yet hard to reconcile with the idea of sharing the same fundamental constitutional rights. Starting with everyone’s birth.

